# Impact of Perinatal Systemic Hypoxic–Ischemic Injury on the Brain of Male Offspring Rats: An Improved Model of Neonatal Hypoxic–Ischemic Encephalopathy in Early Preterm Newborns

**DOI:** 10.1371/journal.pone.0082502

**Published:** 2013-12-06

**Authors:** Yuejun Huang, Huihong Lai, Hongwu Xu, Weizhao Wu, Xiulan Lai, Guyu Ho, Lian Ma, Yunbin Chen

**Affiliations:** 1 Transforming Medical Center, Second Affiliated Hospital of Shantou University Medical College, Shantou, Guangdong, China; 2 Department of Pediatrics, Second Affiliated Hospital of Shantou University Medical College, Shantou, Guangdong, China; 3 Department of Neurosurgery, Second Affiliated Hospital of Shantou University Medical College, Shantou, Guangdong, China; 4 Maternal and Child Health Hospital of Guangdong Province, Guangzhou, Guangdong, China; Robert Debre Hospital, France

## Abstract

In this study, we attempted to design a model using Sprague-Dawley rats to better reproduce perinatal systemic hypoxic-ischemic encephalopathy (HIE) in early preterm newborns. On day 21 of gestation, the uterus of pregnant rats were exposed and the blood supply to the fetuses of neonatal HIE groups were thoroughly abscised by hemostatic clamp for 5, 10 or 15 min. Thereafter, fetuses were moved from the uterus and manually stimulated to initiate breathing in an incubator at 37 °C for 1 hr in air. We showed that survival rates of offspring rats were decreased with longer hypoxic time. TUNEL staining showed that apoptotic cells were significant increased in the brains of offspring rats from the 10 min and 15 min HIE groups as compared to the offspring rats in the control group at postnatal day (PND) 1, but there was no statistical difference between the offspring rats in the 5 min HIE and control groups. The perinatal hypoxic treatment resulted in decreased neurons and increased cleaved caspase-3 protein levels in the offspring rats from all HIE groups at PND 1. Platform crossing times and the percentage of the time spent in the target quadrant of Morris Water Maze test were significantly reduced in the offspring rats of all HIE groups at PND 30, which were associated with decreased brain-derived neurotrophic factor levels and neuronal cells in the hippocampus of offspring rats at PND 35. These data demonstrated that perinatal ischemic injury led to the death of neuronal cells and long-lasting impairment of memory. This model reproduced hypoxic ischemic encephalopathy in early preterm newborns and may be appropriate for investigating therapeutic interventions.

## Introduction

Hypoxic-ischemic encephalopathy (HIE) is the brain manifestation of systemic asphyxia [1,2]. Severe fetal hypoxia in the late gestation is a common event, whether caused by transient interruption of umbilical blood flow, strong uterine contractions, or placental detachment. Acute hypoxic events occurring either in utero during pregnancy or in the period between the start of labor and final expulsion of the fetus remains a significant cause of neonatal HIE [3,4]. Recent data revealed that neonatal HIE affected 2–5 of 1000 live births in the developed countries, with an even higher incidence in the developing countries [5,6]. Neonatal HIE is one of the most important causes of moderate and severe neurological disabilities in children. Furthermore, long-term evaluations have found that there are manifestations of subtle cognitive deficits and behavioral alterations even in cases of mild neonatal HIE [7,8]. However, clinical and pathological features of human perinatal brain lesions display striking variations according to the gestational ages [9]. Variations of brain damage according to gestational age have also been reported in newborn rodents exposed to hypoxic-ischemia (HI) [10,11]. In fact, distinct neuropathological signatures have been noticed depending on HI timing. HI exposure of rats at post-natal day 1 (P1) corresponding to the human early preterm stage of brain development results in multifocal white-matter lesions [10]. In contrast, HI exposure of rats at P7 or P12 corresponding respectively to the late preterm and full-term human brain stages of development results in severe parasagittal cortico-subcortical infarcts [10]. One of the most frequently used models of HI brain damage in immature animals is the Levine model [12] adapted to the 7-day postnatal rat by Rice et al. [13]. Thus, we attempted to establish an animal model which might be better reproduce neonatal HIE in early preterm newborns to investigate therapeutic interventions.

The first goal of this study was to establish a clinically relevant animal model of perinatal systemic hypoxic–ischemic brian damage in early preterm newborns. The second goal of this study was to investigate the effect of fetal hypoxia occurring in the late gestation on immediate changes in brain pathology of neonatal offspring rats [postnatal day (PND) 1]. The third goal was to investigate the long-term effect of fetal hypoxia on the cognitive function of juvenile offspring rats (PND 30 – PND 35).

## Materials and Methods

### Animals

Adult male (n = 15) and female (n = 30) Sprague-Dawley rats (approximately 8 weeks old; 250-300 g and 200-250 g, respectively) were provided by the Animal Center of Shantou University. Female rats were nulliparous. At the start of the experiment,, female rats were randomly divided into 4 groups: control group (n=6), 5 min HIE group (n=6), 10 min HIE group (n=8), 15 min HIE group (n=10). Female rats and male rats were housed in respective pairs in plastic, non-transparent cages (60cm × 40cm × 25cm) under controlled 12-hrs light/12-hrs dark conditions (lights on at 08:00) and temperature (24°C). All rats had free access to food and water throughout the experiments. Rats were acclimated to these conditions for 1 week prior to any experimental procedure. All female rats were housed in pairs with a male for 2 week for mating. The day when sperm was observed in vaginal smears was designated as embryonic day 0 (E 0) [14]. All female rats became pregnant. Nest material was provided for gestational rats, which were housed singly and not disturbed.

All animal experiments were reviewed and approved by the Medical Animals Care and Welfare Committee of Shantou University Medical College (Shantou, China). All studies were carried out in accordance with the US National Institutes of Health Guide for the Care and Use of Laboratory Animals (NIH Publications No. 80-23 revised 1996). Every effort was made to minimize the number of animals used and reduce suffering.

### Neonatal HIE animal model

The pregnant rats at E 21 were anaesthetized with phenobarbital via the caudal vein injection and the hysteras of pregnant rats were exposed by opening the peritoneal cavity. A disposable vascular clamp was placed to the blood vessels adjacent to each placenta to abscise the blood supply to the fetus and the blood vessels were then ligated. The uterine horn were exteriorized and placed on a sterile heated pad at 37 °C for 5, 10 or 15 min. Thereafter, fetuses were rapidly delivered from the uterus, umbilical cords were ligated, and offspring rats were cleaned and manually stimulated to breathe by gentle palpation of the chest and abdomen using moist cotton tips. An offspring rat was considered irrecoverable when no breathing was observed after 5 min of palpation. All live offspring rats were allowed to recover for 1 h in an incubator at 37 °C in air, and then cross-fostered to a dam that had given birth 24 hrs prior. The uterine horns of pregnant rats in the control group were dissected immediately, and then the fetuses were dislodged from the hystera and cross-fostered in the same manner as hypoxic fetuses. The day of delivery was designated as PND 0. In order to reduce suffering and avoid the impact of the sexual dimorphism for hypoxia, we only selected male offspring rats for subsequent experiments. We discriminated the sex of offspring rats at PND 0 by the length between anus and genitalia. The length between anus and genitalia are longer in the males than in the females.

### Survival rate of offspring rats

The survival rate of neonatal rats from different groups at PND 1 was calculated. 12 offspring rats were randomly selected from each group and sacrificed at PND 1 for the western blotting analysis, TUNEL and Nissl staining of brain tissues. 

The remaining offspring rats were cross-fostered to PND 35. The condition of offspring rats were monitored every 4 hrs during the first week of the birth and extended to 12 hrs a thereafter. Offspring rats were euthanasia by carbon dioxide asphyxiation when they exhibited signs of no moving or eating, weak breathing, convulsion, cold limbs and cyanosis. The Log-rank test was used to compare survival rats among groups at PND 35.

### Brain tissue samples of offspring rats at PND 1

12 offspring rats from each group were killed at PND 1 by decapitation after anaesthetized with sodiumpentobarbital (50 mg/kg, i.p.). Brains of the offspring rats were rapidly removed on ice and cleaned with ice-cold heparinized 0.1 M phosphate buffer solution (PBS). The whole brain was cut longitudinally into hemicerebrum. The right side of hemicerebrum were frozen in liquid nitrogen and stored at -70°C for western blotting analysis. The left side of hemicerebrum was used for TUNEL and Nissl staining. Brain tissues (including cortex and hippocampus, approximately 3-4mm) used for TUNEL and Nissl staining were placed in 4% paraformaldehyde in 0.1 M phosphate buffer (pH 7.4). After 24 hrs of fixation, the tissues were embedded in paraffin and cut to 5 um and 3 um thick sections for TUNEL staining and Nissl staining, respectively. 

### TUNEL staining

TUNEL staining was performed on paraffin-embedded sections by using the in situ cell death detection kit (Roche Applied Science, Mannheim, Germany) according to the manufacturer’s instructions. Briefly, sections were deparaffinized in xylene, rehydrated through graded ethanol, rinsed in 3% hydrogen peroxide and treated with proteinase K (20 mg/ml) for 25 min at room temperature. Subsequently, the sections were incubated with the TUNEL reaction mixture for 1 h at 37 °C. After washing with PBS, the sections were incubated with coverter-POD for 30 min and then visualized with DAB (Maixin, Fujian, China). Sections were then counterstained with hematoxylin for 3 min and rinsed under running water. After dehydration in graded ethanol series and transparent in xylene, the brain sections were mounted onto gelatin-coated slides. 

Cells with yellow-brown granules in the nucleus were considered to be apoptotic cells. Counting of apoptotic cells was performed in the CA1 area of hippocampus and cerebral cortex. Total TUNEL positive stained cells were counted in six randomly chosen views (three views in hippocampus and three views in cerebral cortex) under the light microscope with 400× magnification for each section and the mean number was derived per section. The average number of apoptotic cells of eight sections from each sample was calculated to assess the severity of the brain damage. The cell count was performed by an investigator who was blinded to the experimental protocol.

### Morris Water Maze Test

The cognitive function of offspring rats were evaluated using Morris water maze test at PND 30. The method of Morris water maze test was described by us previously [15]. The water maze was a black circular pool (160 cm in diameter) conceptually divided in four equal quadrants for the purpose of data analysis. The water temperature was maintained between 22–24 °C. There was a black circular platform of 12 cm in diameter at two centimeters beneath the water surface and hidden from the rats. It had a rough surface, which allowed the rat to climb onto it easily once detected. The swimming path of the rats was recorded using a video camera mounted above the center of the pool and analyzed using a video tracking and analysis system (product of the Institute of Materia Medica, Chinese Academy of Medical Sciences). The water maze was located in a well-lit white room with several posters and other distal visual stimuli hanging on the walls to provide spatial cues. A curtain separated the water maze room from the room where the computer setup was installed. 

A 5-day training-test procedure was employed. On each training day, the rats received four training sections while the hidden platform was kept in constant position. In each section, rats were released into the pool from one of the four different quandrants along the wall, and the performance of the rat was monitored by a CCD camera/image analyzer. The time required to reach the platform was recorded. If a rat could not find the platform within 60 s after release, it was led to the platform and placed on it for 20 s before being removed. In these cases, escape latency (EL) of 60 s was recorded. We conducted the trial once a day for four days for each rat. On the fifth day, a probe test, as a trial without the platform, was conducted; in this procedure, the test parameters included swimming track, the time reaching the platform, swimming distance of the platform space, the time spending in the target quadrantand and platform crossing times.

### Brain tissue samples of offspring rats at PND 35

The offspring rats at PND 35 were anaesthetized with sodiumpentobarbital (50 mg/kg, i.p.), killed, and brains rapidly removed. The left side of hippocampus was placed in 4% paraformaldehyde in 0.1 M phosphate buffer (pH 7.4) for Nissl staining. The right side of hippocampus were frozen in liquid nitrogen, and stored at -70 °C for western blotting analysis.

### Nissl staining

After 24 hrs of fixation, the tissues were embedded in paraffin, and then cut into 3um thick sections. After dewaxing in xylene and rehydration through graded ethanol, the sections were hydrated in 1% (w/v) toluidine blue at 37 °C for 20 min. After rinsing with double distilled water, they were dehydrated and mounted with permount. 8 slices per brain were used for cell counting. Six fields of each slice in the hippocampus (CA1) or cortex were chosen randomly at 400x magnification to count staining cells. Imaging-Pro-Plus software 6.0 was used to perform quantitative analysis of cell number count. The mean number of intact neurons in the six views was used for cell count in each section. The average number of eight sections from each sample was used for analysis. Nissl-positive cell count was performed by an investigator who was blinded to the experimental protocol.

### Western blot analysis

Brain tissues were homogenized in cold RIPA lysis buffer (Beyotime, Jiangsu, China) and the protein concentrations were determined by BCA assay kits (Beyotime, Jiangsu, China). Equal amounts of protein (60 μg) were separated on 12% SDS-PAGE and transferred onto polyvinylidene fluoride (PVDF) membrane (pore size, 0.22 μm; Millipore). The membrane was blocked in a Tris-buffered saline solution (TBS-T: 20 mM Tris, pH 7.6, 135 mM NaCl, and 0.05% Tween) containing 5% nonfat dry milk at room temperature for 1hr, and then incubated with different primary antibodies including anti- brain-derived neurotrophic factor (BDNF) (1:800, Biosynthesis, Beijing, China), anti-β-actin(1:1500, Biosynthesis, Beijing, China) overnight at 4°C. After washing the membrane 3x with TBS-T, the secondary antibody HRP-labeled goat-anti-rabbit IgG was added to the membrane according to the vendor’s recommendation (1:8000, Biosynthesis, Beijing, China) and incubated for 1 h at room temperature. The membrane was then washed again as described previously. The bound antibodies were detected by using SuperSignal western blotting kits. Quantity One Software (v4.5.2, Bio-Rad, Hercules, CA, USA) was used to perform densitometric analysis of western blots. The relative protein level of BDNF was calculated relative to that of β-actin.

Protein levels of procaspase-3 and the cleaved caspase-3 were analyzed by western blots as described above. 12% and 15% SDS-PAGE gels were used for procaspase-3 and cleaved-caspase-3, respectively. The primary antibodies used were anti-procaspase-3 (1:1000, Biosynthesis, Beijing, China) and anti-cleaved-caspase-3 (1:1000, Biosynthesis, Beijing, China).

### Statistical analysis

Each experiment was performed at least three times. Statistical analyses were performed using SPSS 17.0 (SPSS Inc., Chicago, IL,USA).The results are expressed as mean ± S.D.. One-way analysis of variance (ANOVA) followed by Student–Newman–Keuls’s test was used to compare the differences among multiple groups. Log-rank test were done to compare the survival curves among groups. Data of EL from four training days in the Morris Water Maze test were analyzed using the general linear models repeated-measures analysis of variance. The correlations among the data in the Morris water maze test, BDNF levels, and Nissl-postive cells in the hippocampus of offspring rats was analyzed by Bivariate correlations. Statistical significance was set at *P* < 0.05.

## Results

### Survival rate of male offspring rats at PND 1 and PND 35

**Figure 1:**
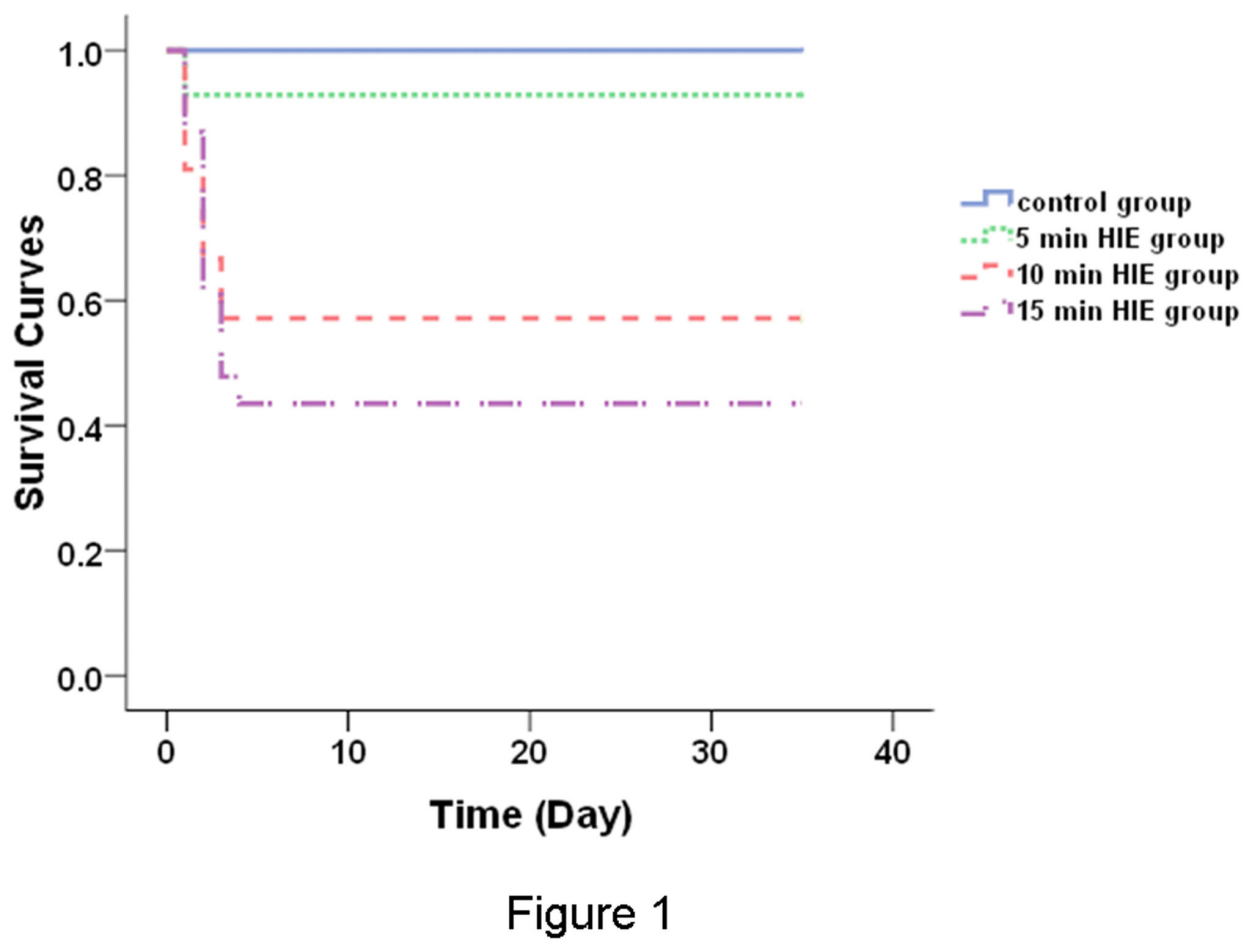
Survival curves of neonatal offspring rats from different groups. The survival curves of offspring rats were displayed at PND 35 (control group, n=12; 5 min HIE group, n= 14; 10 min HIE group, n= 21; 15 min HIE group, n= 24).

In this study, there were 161 male fetuses from 30 maternal rats. Offspring rats from the control group were successfully recovered from the caesarean delivery. The survival rates of male offspring rats at PND 1 for control, 5 min, 10 min, and 15 min HIE groups were 100% (24 of 24), 86.7% (26 of 30), 70.2% (33 of 47), 58.3% (35 of 60), respectively, indicating a makred reduction of survivals of male offspring rats during the 10-minute hypoxic treatment. As shown in Figure 1, the survival rate of male offspring rats at PND 35 for control, 5 min HIE, 10 min HIE, and 15 min HIE groups were 100% (12 of 12), 92.9% (13 of 14), 57.1% (12 of 21), 43.5% (10 of 23), respectively. The Log-rank test revealed that the survival rate of male offspring rats at PND 35 from difference groups were control group > 5 min HIE group > 10 min HIE group > 15 min HIE group (Chi-Square = 15.344, df = 3, *P* = 0.002).

### Neuronal cells in the brains of offspring rats at PND 1

**Figure 2:**
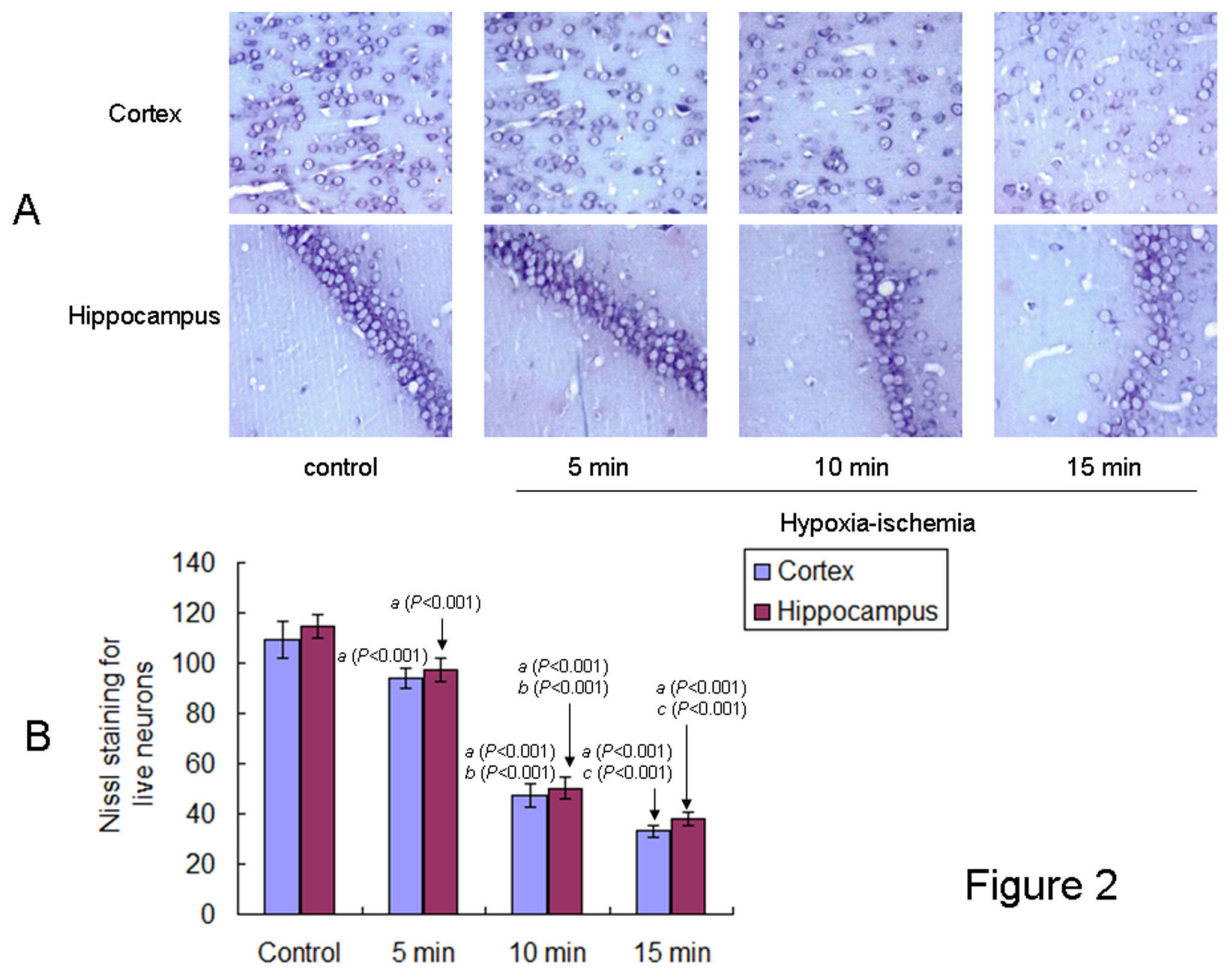
Nissl staining of neuronal cells in the brain of neonatal offspring rats from different groups. **A**: Representative photographs of Nissl staining of neurons in the cortex and hippocampus (CA1) of neonatal rat brains from different groups were showed (scale bar: 400μm). **B**: Nissl-positive neuronal cells were counted in six randomly chosen views. Total Nissl-positive cells per view in the cortex and hippocampus (CA1) of offspring rats from different groups (n= 12 in each group) at PND 1 were showed. The results were expressed as mean ± S.D.. *a*: compared with the control group; *b*: compared with the 5 min HIE group; *c*: compared with the 10 min HIE group. All *P* values were <0.001.

Neuronal cells in both the cortex and hippocampus of offspring rats at PND 1 were identified by Nissl staining (Figure 2A). As expected, HIE significantly reduced the number of neuronal cells and resulted in smaller and irregularly arranged neurons in the brain of offspring rats from all HIE groups as compared to the offspring rats in the control group at PND 1 (Figure 2B). The decrease was most pronounced in the 15 min HIE group, followed by the 10 min and 5 min HIE groups [cortex: F (3,44) = 577.429, *P* < 0.001, ANONA; hippocampus: F (3,44) = 847.32, *P* < 0.001, ANONA].

### Apoptosis cells in the brains of offspring rats at PND 1

**Figure 3:**
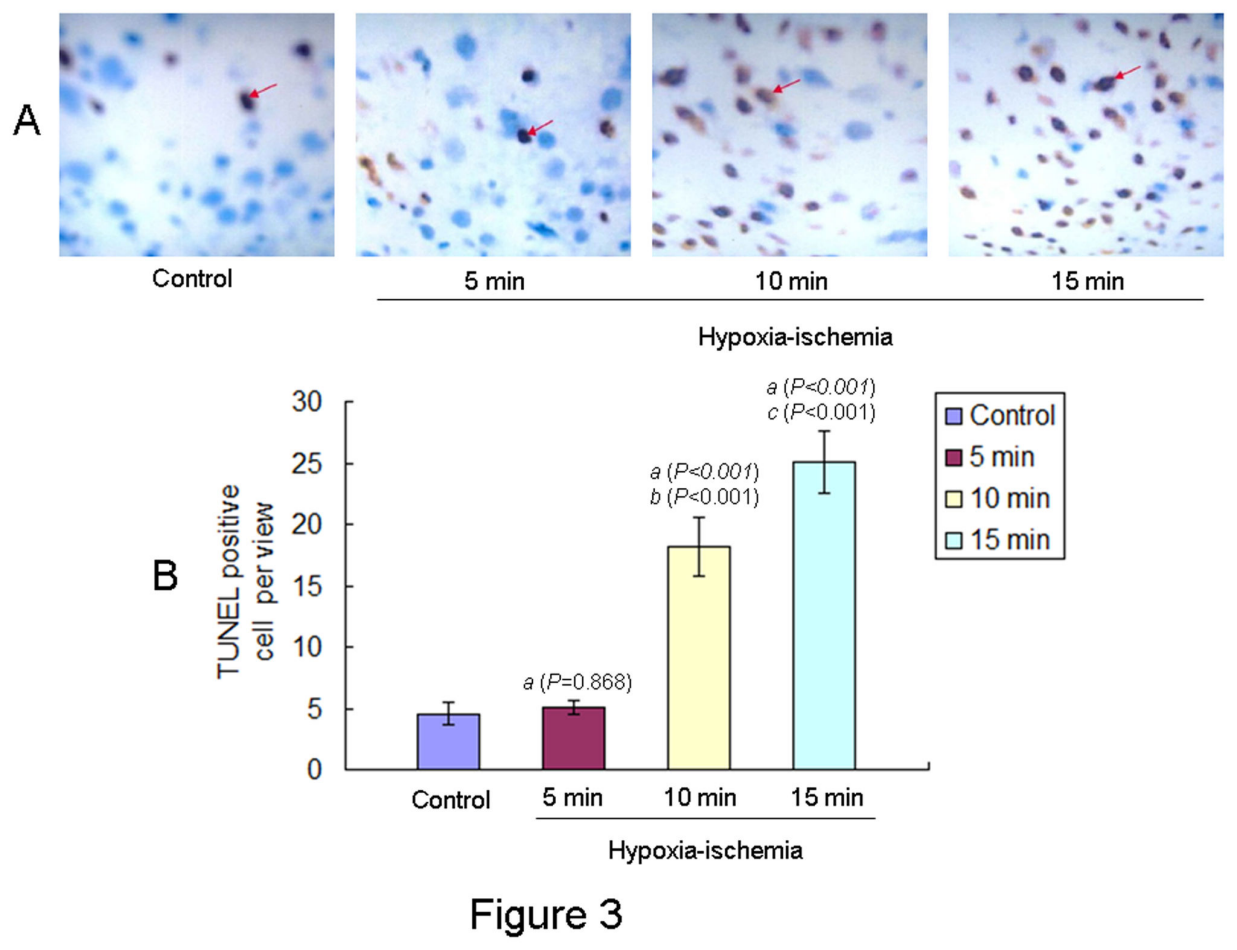
TUNEL staining of apoptotic cells in the brains of neonatal offspring rats from different groups. **A**: Apoptotic cells were detected by TUNEL staining (dark brown, indicated by arrows) in the brains of offspring rats at PND 1 after ischemic injury. Scale bar: 400μm. **B**: Total TUNEL-positive cells were calculated in six randomly chosen views (three in hippocampus and three in cerebral cortex). Total TUNEL-positive cells per view from different groups (n=12 in each group) at PND 1 were showed. The results were expressed as mean ± S.D.. *a*: compared with the control group; *b*: compared with the 5 min HIE group; *c*: compared with the 10 min HIE group. The P values were showed in the bracket.

Apoptotic cells in the brain of offspring rats on PND 1 after ischemic injury were detected using TUNEL staining (Figure 3A). Total TUNEL-positive cells per view were showed in Figure 3B. In the 5 min HIE group and control group, there were few apoptotic cells and the difference between the two groups was not statistically significant. Apoptotic cells were significant increase in the 10 min and 15 min HIE groups compared to the control and there were greater numbers of apoptotic cells in the 15 min HIE group than those in the 10 min HIE group (Figure 3B). One way ANOVA with the number of apoptotic cells as the dependent variable and HIE as the fixed factor revealed an effect of HIE [F(3,44) = 371.886, *P* < 0.001] on the apoptotic cells in the brains of offspring rats.

### Relative levels of cleaved caspase-3 to procaspase-3 in the brains of offspring rats at PND 1

**Figure 4:**
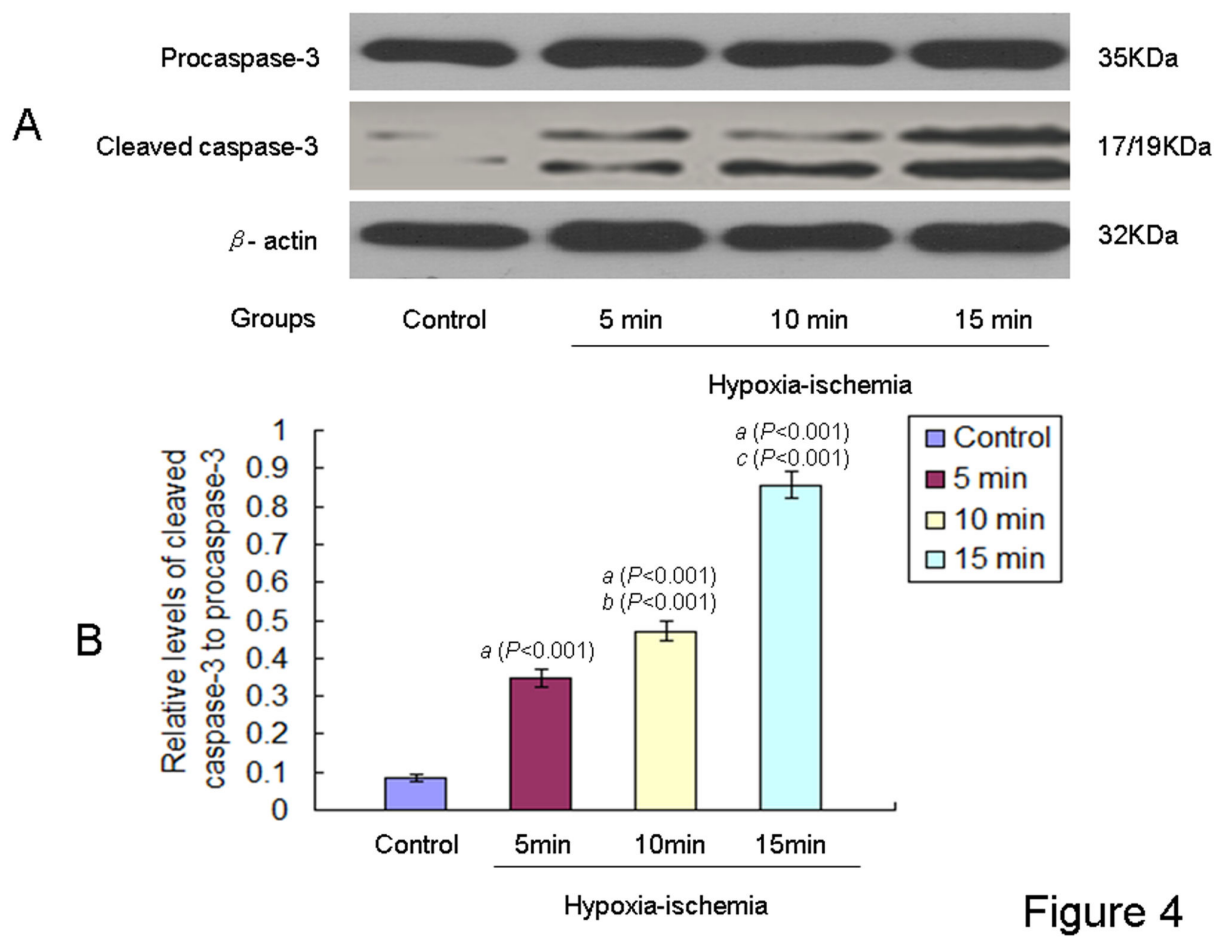
Levels of cleaved caspase-3 to procaspase-3 in the brain of neonatal offspring rats from different groups. **A**: Representative Westernblots of cleaved caspase-3 and procaspase-3 from brain tissues of offspring rats with or without ischemic injuries were showed. **B**: Levels of cleaved caspase-3 to procaspase-3 in the brain of neonatal offspring rats from different groups (n= 12 in each group) at PND 1 were calculated relative to that of β-actin. The results were expressed as mean ± S.D.. *a*: compared with the control group; *b*: compared with the 5 min HIE group; *c*: compared with the 10 min HIE group. All *P* values were <0.001.

Protein levels of β-actin, cleaved Caspase-3, and procaspase-3 were detected in the brains of offspring rats (Figure 4A). As can be seen in Figure 4B, the brain ischemic injury resulted in increased levels of cleaved caspase-3 relative to procaspase-3 in the offspring rats from all HIE groups at PND 1. Quantitative analysis by densitometry scan showed that levels of cleaved caspase-3 relative to procaspase-3 were most pronounced in the 15 min HIE group, followed by the descending order of 10 min, 5 min, and control groups [F (3,44) = 1938.485, *P* < 0.001, ANONA].

### Effects of neonatal HIE on memory function of offspring rats at PND 30

**Figure 5:**
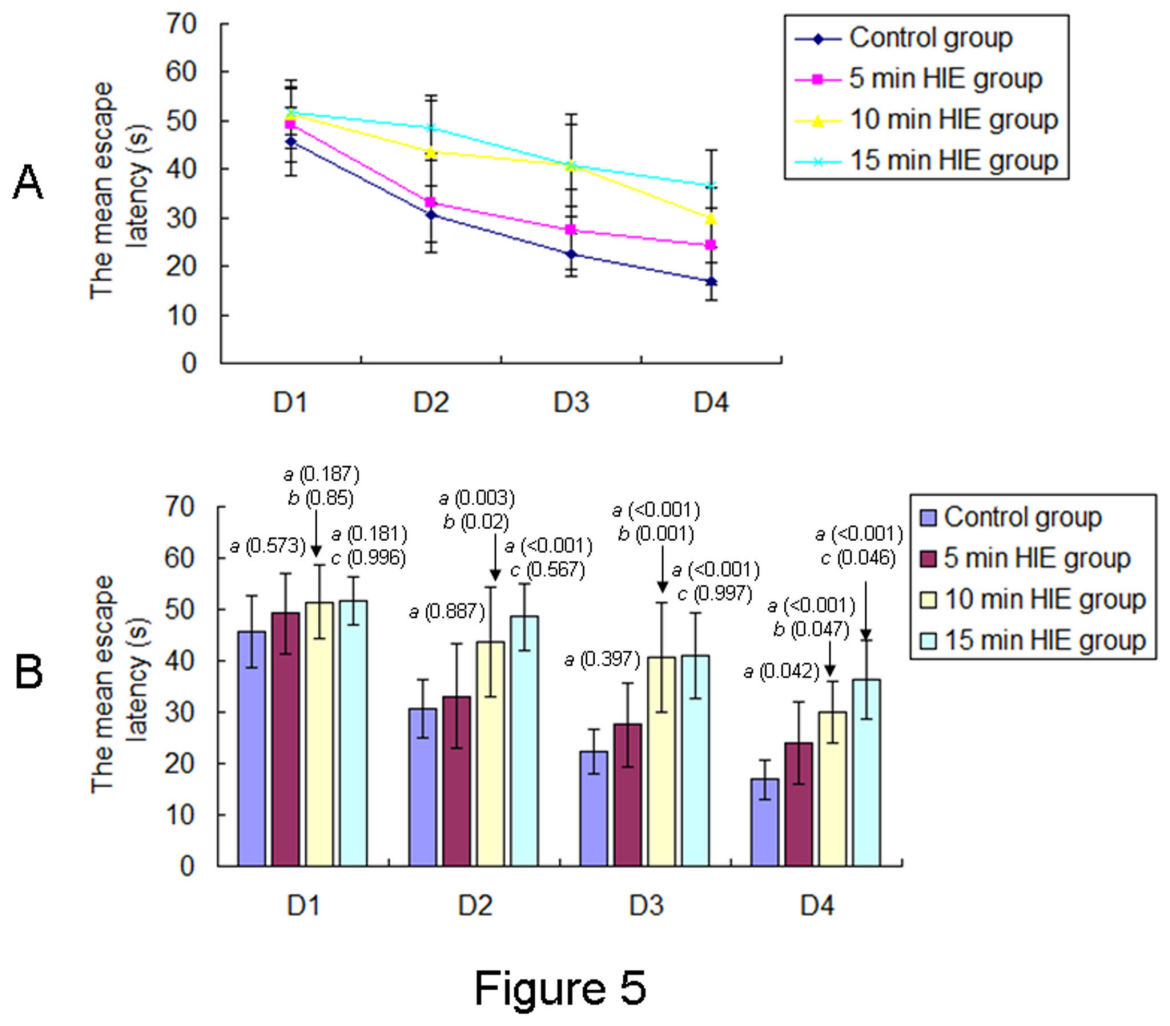
The mean EL of offsprings over 4-day training periods in the Morris water maze test. **A**: The learning curves for the offspring rats from different groups over the course of 4-day training periods in the Morris water maze test were showed. **B**: The mean EL of the offspring rats from different groups (control group, n= 12; 5 min HIE group, n= 13; 10 min HIE group, n= 12; 15 min HIE group, n= 10) were expressed as mean ± S.D.. *a*: compared with the control group; *b*: compared with the 5 min HIE group; *c*: compared with the 10 min HIE group. The P values were showed in the bracket.

Figure 5A shows the learning curves for the mean escape latencies of offspring rats over the course of 4-day training periods in the Morris water maze test. One-way repeated ANOVA with HIE as the independent factor and time as the repeated factor revealed the effect of HIE on the mean escape latencies [F(3,43) = 20.803, *P* < 0.001] of offspring rats in the Morris Water Maze test. Mean escape latencies were similar for the offspring rats in all groups on the first day of training. On the second and third days, the escape latency of the offspring rats in the 10 min and 15 min HIE groups was significantly longer than those in the 5 min HIE and control groups. By the fourth day, the escape latency of the offspring rats in all HIE groups was significantly longer than that in the control group (Figure 5B). One way ANOVA with the mean escape latencies as the dependent variable and HIE as the fixed factor revealed an effect of HIE [D1 : F(3,43) = 1.924, *P* = 0.14; D2 : F(3,43) = 10.889, *P* < 0.001; D3 : F(3,43) = 15.259, *P* < 0.001; D4 : F(3,43) = 17.834, P < 0.001] on the mean escape latencies of offspring rats in the Morris Water Maze test. 

**Figure 6:**
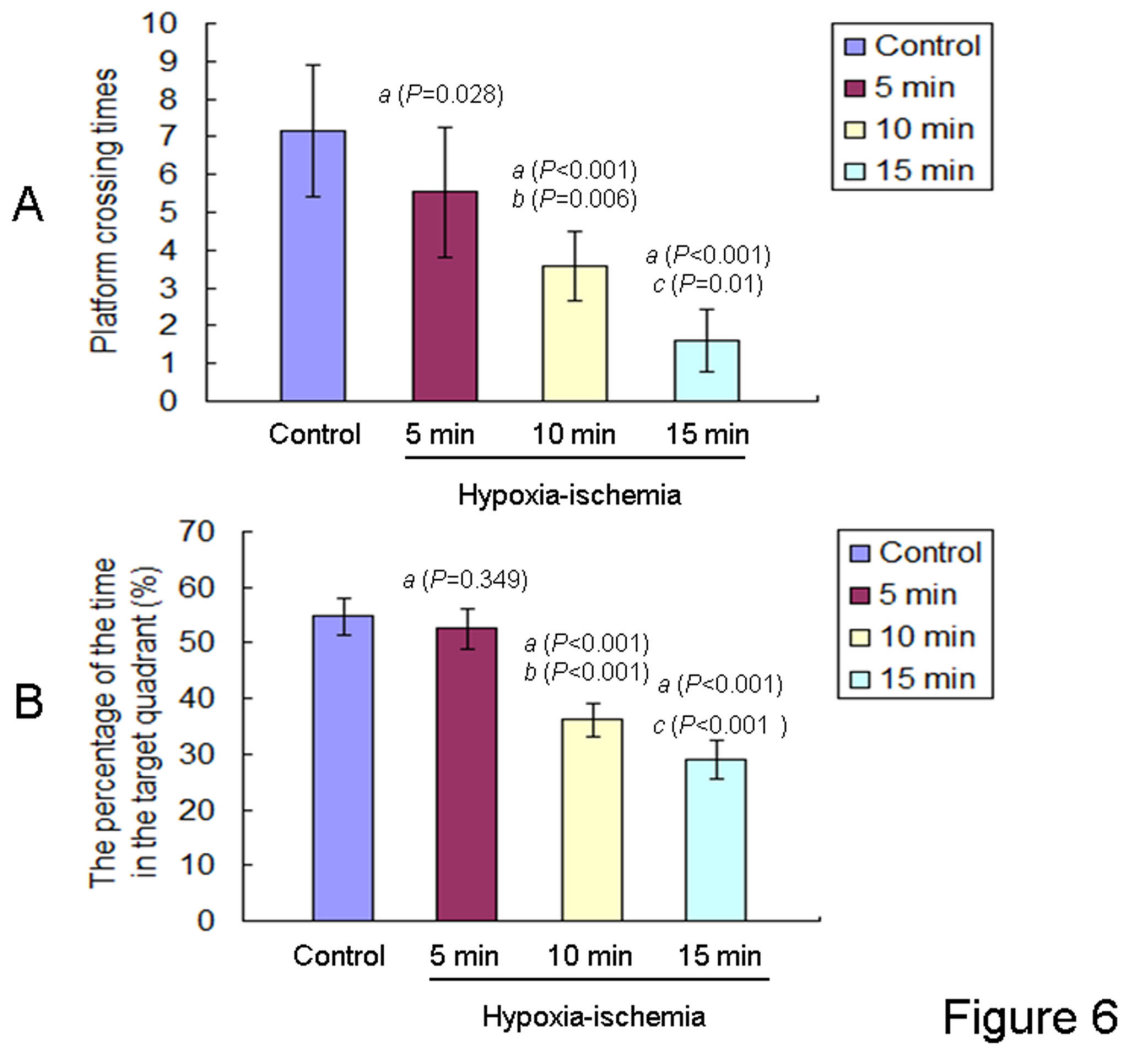
Platform crossing times and the target quadrant time of offspring rats in Morris water maze test. **A**: The platform crossing times of the offspring rats from different groups (control group, n= 12; 5 min HIE group, n= 13; 10 min HIE group, n= 12; 15 min HIE group, n= 10) were expressed as mean ± S.D.. *a*: compared with the control group; *b*: compared with the 5 min HIE group; *c*: compared with the 10 min HIE group. The P values were showed in the bracket. **B**: The percentage of the time spent in the target quadrant of offspring rats from different groups (control group, n= 12; 5 min HIE group, n= 13; 10 min HIE group, n= 12; 15 min HIE group, n= 10) were expressed as mean ± S.D.. *a*: compared with the control group; *b*: compared with the 5 min HIE group; *c*: compared with the 10 min HIE group. The P values were showed in the bracket.

In the probe test, where the platform was removed,, the offspring rats in the control group rapidly found the position where the platform had been, and their escape latencies did not differ from their previously acquired level. In contrast, the offspring rats in all HIE groups were impaired in locating the platform where it had been and they reverted to their mean baseline latencies. Platform crossing times and the percentage of the time spent in the target quadrant were showed as Figure 6A and 6B. The number of times the rat crossed the position where the platform had been were significantly reduced in the offspring rats of all HIE groups as compared to the control group. The reduction of cross times was most pronounced in the 15 min HIE group, followed by the 10 min and 5 min HIE groups as compared to the control group [F (3,43) = 32.83, *P* < 0.001, ANONA]. The offspring rats in the 15 min and 10 min HIE groups spent less time in the target quadrant compared with the control group. No significant differences were identified in the offspring rats between the 5 min HIE and control groups. One way ANOVA with the percent time in quadrant as the dependent variable and HIE as the fixed factor revealed an effect of HIE [F(3,43) = 160.369, P < 0.001] on the percentage of the time spent in the target quadrant of offspring rats in the Morris Water Maze test.

### Neuronal cells in the brains of offspring rats at PND 35

**Figure 7:**
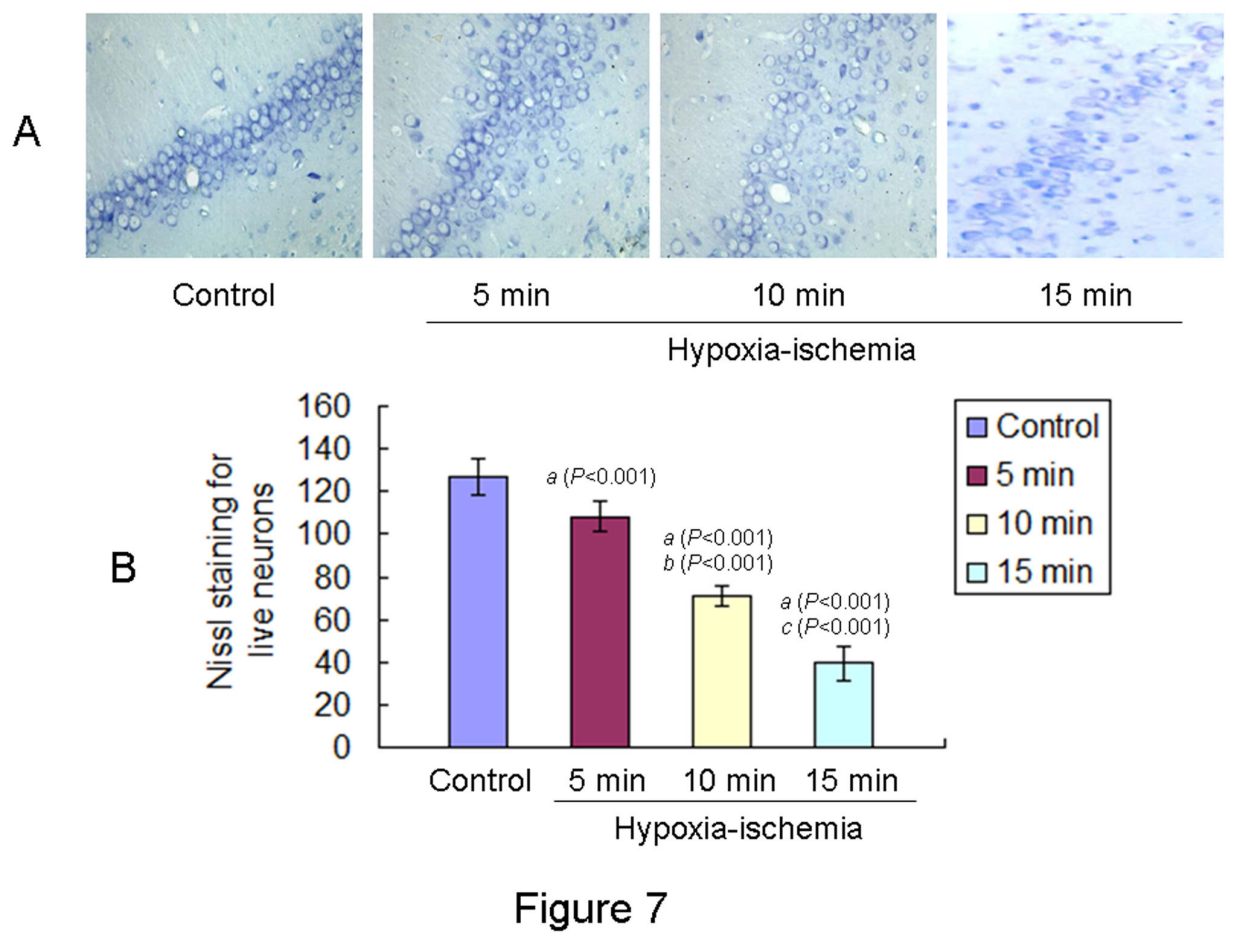
Nissl staining showed neuronal cells in the hippocampus of offspring rats from different groups. **A**: Representative histological sections of Nissl staining of neurons in the hippocampus (CA1) of neonatal rat brains from different groups were showed (scale bar: 400μm). **B**: Total Nissl-positive neuronal cells were calculated in six randomly chosen views. Total Nissl-positive neuronal cells per view in the hippocampus (CA1) of offspring rats from different groups (n= 12 in each group) at PND 35 were showed. The results were expressed as mean ± S.D.. *a*: compared with the control group; *b*: compared with the 5 min HIE group; *c*: compared with the 10 min HIE group. All *P* values were <0.001.

Neuronal cells in the hippocampus of offspring rats at PND 35 were identified by Nissl staining (Figure 7A). Live neuronal cells were significant decrease in the brain of offspring rats from all HIE groups as compared to the offspring rats in the control group (Figure 7B). The decrease in the number of live neuronal cells per view was most pronounced in the 15 min HIE group, followed by 10 min and 5 min HIE groups as compared to the control [F (3,43) = 794.231, *P* < 0.001, ANONA].

### Effects of neonatal HIE on the expression of BDNF in the hippocampal of offspring rats at PND 35

**Figure 8:**
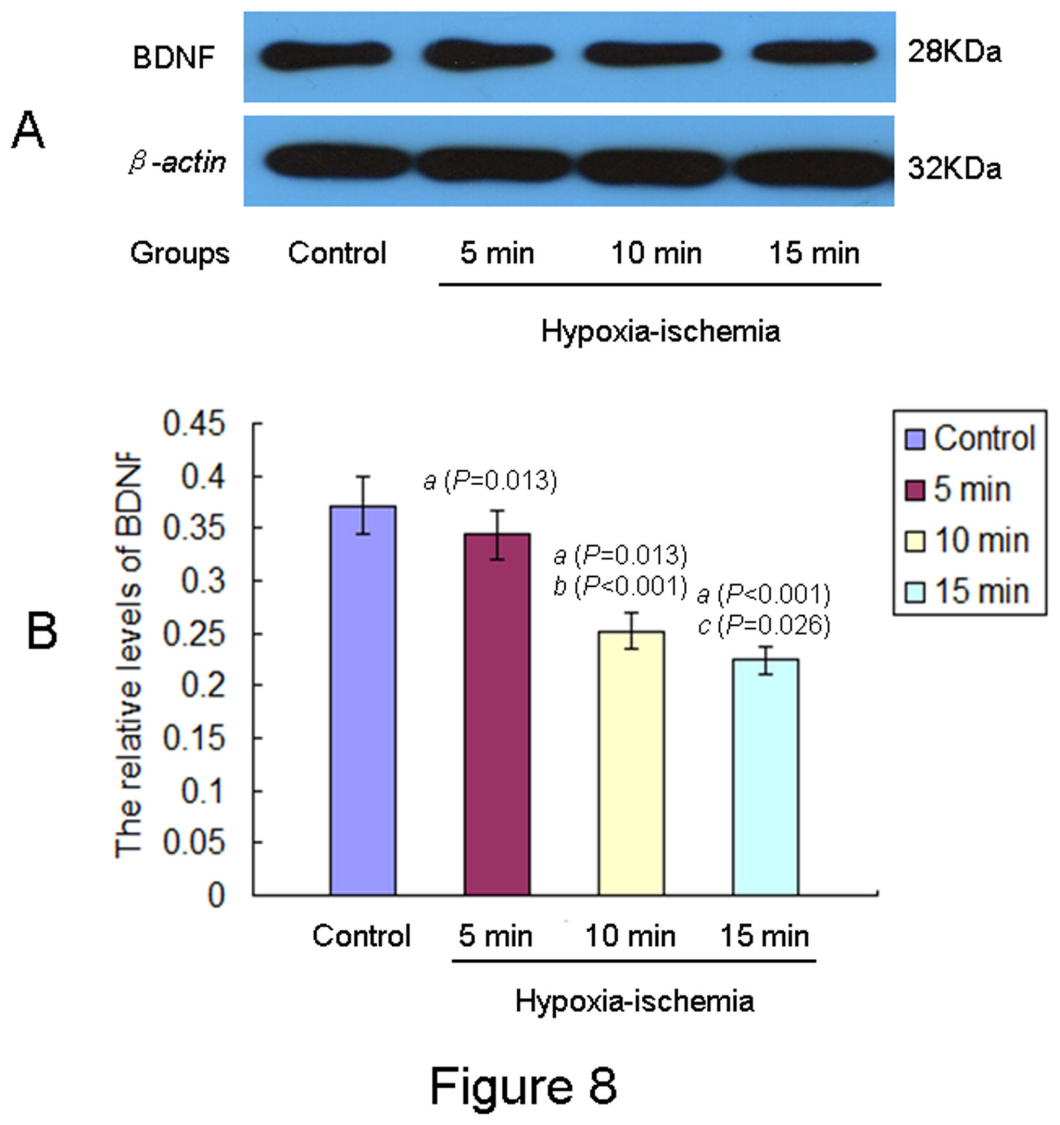
Expression of BDNF in the hippocampal of offspring rats from different groups. **A**: A representative Western blot of BDNF protein in the hippocampal of offspring rats from different groups was showed. **B**: Relative levels of BDNF in the hippocampal of offspring rats from different groups (control group, n= 12; 5 min HIE group, n= 13; 10 min HIE group, n= 12; 15 min HIE group, n= 10) at PND 35 were expressed as mean ± S.D.. *a*: compared with the control group; *b*: compared with the 5 min HIE group; *c*: compared with the 10 min HIE group. The P values were showed in the bracket.

Protein levels of β-actin and BDNF could be detected in the hippocampus of offspring rats (Figure 8A). The relative levels of BDNF to β-actin were lower in the hippocampus of offspring rats in all HIE groups than in the control group (Figure 8B). The decreased trend for the relative levels of BDNF to β-actin were 15 min HIE group < 10 min HIE group < 5 min HIE group < control group [F (3,43) = 123.885, *P* < 0.001, ANONA).

### Correlations among the data in the Morris water maze test, BDNF levels, and live neuronal cells in the hippocampus of offspring rats at PND 35

Bivariate correlations demonstrated correlations among the data in the Morris water maze test, BDNF levels, and live neuronal cells in the hippocampus of offspring rats at PND 35. Offspring rats with less live neuronal cells have decreased BDNF expression (r = 0.861, *P* < 0.001) in the hippocampus also exhibited less platform crossing times (*r* = 0.839, *P* < 0.001) and less time spent in the target quadrant (*r* = 0.868, *P* < 0.001) in the Morris water maze test.

## Discussion

There was a commonly used animal model for perinatal asphyxia in early preterm newborns, in which the pregnant rats were sacrificed first, thus leading to fetal hypoxia as a result of death of the mother [16,17]. Our study attempts to better reproduce this phenomenon by abscising the placenta blood supply to the fetuses, thus avoiding the deaths of maternal rats. In addition, we performed comprehensive assessments of the offspring rats, including survival studies, the sign of apoptosis, behavioral analysis and the associated mechanistic studies.

The biochemical cascade of events initiated by hypoxia is complex. Depletion of cellular energy reserves is a major problem [18] and the mitochondrial response to fetal hypoxia is critical in determining neuronal cell fate [19]. Severe tissue oxygen depletion could cause total mitochondrial failure and necrotic cell death, while less severe oxygen deprivation any trigger activation the apoptotic pathway [20]. Apoptosis, first characterized by Kerr et al. [21], is a programmed cell death defined by the nuclear and cell morphological changes resulting from activation of its signaling cascades. 

Caspases are a superfamily of cysteine aspartylspecific proteases that regulate many aspects of cell survival and death [22]. Caspase-3 is a validated marker in detecting early neuronal apoptosis [22,23]. Its activation is mediated both via extrinsic (death ligand) and intrinsic (mitochondrial) pathways [24]. The zymogen caspase-3 has virtually no activity until it is cleaved by an initiator caspase after apoptotic signaling events have occurred [25]. Caspase-3 is specifically activated in neuronal cell bodies and their processes following HI [26]. Caspase-3 activation occurs in striatum and hippocampus at 12–18h after HI [27]. 

In this study we reported the cleaved caspase-3 protein levels increased in a time-dependent manner in the HIE groups at PND1. In order to corroborate the caspase-3 findings, we also used Nissl staining to identify neuronal cells and TUNEL to detect apoptotic cells. TUNEL detects early-stage apoptosis looking at the initial changes in chromatin condensation, before the nucleus undergoes major morphological changes [28]. In our present study, the cleaved caspase-3 protein levels, TUNEL and Nisll staining demonstrated that peripartal ischemic could induce neuronal apoptosis in the brain of offspring rats at PND 1, and revealed that significant acute brain injury in this animal model.

Long-term cognitive dysfunction is one of the most significant implications of neonatal HIE; yet, clear understanding of the association between morphological changes, apoptosis and cognitive dysfunction remains elusive. Morris water maze test is the standard technique in assessing spatial learning and memory [29] and allows repetitive testing to assess longterm neurocognitive dysfunction following HI injury [12]. Here we report functional impairment at PND 30 after prolonged (>10min) HI injury; rats after 10 and 15min asphyxia required a longer time to reach the hidden platform and spent less time in the platform area during the probe trial. Nevertheless, as we did not report any difference in the total swimming path or the swimming speed for the offspring rats in the Morris water maze test, it is unlikely that the motor deficit contributed to poorer performance in the 10 min and 15 min HIE groups. 

In the present study, we detected the live neuronal cells in the hippocampus of offspring rats at PND 35. We found there were significant positive correlations between live neuronal cells and BDNF expression in the hippocampus. These findings suggest that the reduction of hippocampal BDNF expression in the HIE offspring may be related to the decreased live neuronal cells. Furthermore, delayed neuronal death following perinatal asphyxia injury in rats has also been described by other researchers [30], with neuronal cells loss observed in the CA1 area of the hippocampus for up to 3 months following asphyxia [31].

BDNF is a member of the neurotrophin family. BDNF could affect neuronal survival and differentiation, and play a role as mediators of activity-dependent brain plasticity as well as in spatial learning and memory [32,33]. Consistently, our data demonstrate that neonatal HIE impairs BDNF expression in the hippocampus of offspring rats. Significant correlations were observed between the expression of BDNF and memory function of offspring rats in our study. Rats with lower BDNF expression in the hippocampi exhibited less platform crossing times and less time spent in the target quadrant in the Morris water maze test. These findings suggest that neonatal HIE could cause long-lasting changes in the hippocampi of offspring rats. 

Cerebral palsy and mental retardation are more common in males than in females [34]. While sexual dimorphism has long been recognized in adult stroke both in humans and animal models [35], it has not drawn much attention in animal experiments in neonatal HIE until the last decade. Most research groups designed studies without sex stratification in neonatal HI [36,37]. In practice, clinicians in NICUs have long been aware that baby boys and girls are quite different with regard to susceptibility to various diseases and immaturity. Therefore, in order to avoid the impact of the sexual dimorphism for hypoxia, we only selected male offspring rats for this study. And we will address sex differences in the impact of hypoxia-ischemic injury for offspring rats in our forthcoming studies.

## Conclusion

Our data demonstrated that perinatal hypoxia leads to neuronal cells damage and apoptosis in the brains. Meanwhile, we found cellular changes in neonates were associated with subsequent neurocognitive abnormality in juvenile rats (PND 30). Our model has the advantage of exactly mimicking the process leading to HIE, in which the insult occurs in utero during labor and results in global brain injury. This model might be better suited to investigate the molecular events following the HIE injury and enable testing of novel therapeutic strategies to prevent and treat the HIE in early preterm newborns.
