# Correction: Impact of Perinatal Systemic Hypoxic–Ischemic Injury on the Brain of Male Offspring Rats: An Improved Model of Neonatal Hypoxic–Ischemic Encephalopathy in Early Preterm Newborns

**DOI:** 10.1371/annotation/36f9cc71-3d10-4dcf-8aae-b9224447ce18

**Published:** 2013-12-20

**Authors:** Yuejun Huang, Huihong Lai, Hongwu Xu, Weizhao Wu, Xiulan Lai, Guyu Ho, Lian Ma, Yunbin Chen

The seventh and eighth authors are listed out of order. The correct order is: Yuejun Huang, Huihong Lai, Hongwu Xu, Weizhao Wu, Xiulan Lai, Guyu Ho, Yunbin Chen, Lian Ma. The correct order of the Corresponding Authors' email addresses is: yunbin_chen@hotmail.com (YC); malian8965@sina.com (LM). 

